# Antiparallel RNA G-quadruplex Formed by Human Telomere RNA Containing 8-Bromoguanosine

**DOI:** 10.1038/s41598-017-07050-w

**Published:** 2017-07-27

**Authors:** Chao-Da Xiao, Takumi Ishizuka, Yan Xu

**Affiliations:** Division of Chemistry, Department of Medical Sciences, Faculty of Medicine, University of Miyazaki, 5200 Kihara, Kiyotake, Miyazaki, 889-1692 Japan

## Abstract

In this study, by combining nuclear magnetic resonance (NMR), circular dichroism (CD), liquid chromatography-electrospray ionization-mass spectrometry (LC-ESI-MS), and gel electrophoresis, we report an unusual topological structure of the RNA G-quadruplex motif formed by human telomere RNA r(UAGGGU) containing 8-bromoguanosine. Results showed that the RNA sequence formed an antiparallel tetramolecular G-quadruplex, in which each pair of diagonal strands run in opposite directions. Furthermore, guanosines were observed both in syn- and anti-conformations. In addition, two of these G-quadruplex subunits were found to be stacking on top of each other, forming a dimeric RNA G-quadruplex. Our findings provide a new insight into the behavior of RNA G-quadruplex structures.

## Introduction

G-quadruplexes are higher-order DNA and RNA structures. Structure and function of G-quadruplexes have become an area of great interest. Recently, they have been associated with some human genetic neurodegenerative diseases such as amyotrophic lateral sclerosis (ALS) and frontotemporal dementia (FTD)^1, 2^. G-quadruplexes are also believed to be important for telomere maintenance, involved in cell aging or death mechanisms^3^. Their structure has recently become an attractive therapeutic target for drug design^3–8^. Both X-ray crystallography and NMR have revealed their high resolution structures^9, 10^. The topologies of G-quadruplexes can be classified into several types: antiparallel, parallel, and hybrid G-quadruplex, among others^11–20^. Four guanines in a plane bind to each other through Hoogsteen hydrogen bonds, forming the G-tetrad. Usually, the syn/anti-glycosidic conformation of guanines is considered to be an important factor in the G-quadruplex structure folding.

Contrary to their DNA counterparts, RNA G-quadruplexes are less diverse in terms of stem strand orientations. Recently, a crystal structure study of a RNA aptamer revealed that the RNA folds into a new RNA G-quadruplex motif with two groups of loops in different orientations^21^. According to the crystallographic analysis, the unprecedented complex loops, and not the G-stem itself, were described as “non-parallel”. A recent study tried to govern the formation of a forced RNA antiparallel orientation in G-stem, suggesting that the RNA G-quadruplex was difficult to control in an antiparallel orientation in G-stem than the DNA G-quadruplex^22^. Until now, the studies on RNA G-quadruplexes showed that the RNA sequences can only adopt parallel G-quadruplex. For example, we and other groups demonstrated that human telomere RNA, a newly found telomeric repeat-containing RNA, can fold in a variety of ways to form parallel G-quadruplexes^23–31^.

There has not been any consistent explanation of why RNA sequences cannot form antiparallel G-quadruplexes, although some studies attribute it to the anti-glycosidic conformation of guanosine (rG) and the C3′-endo conformation of sugars^32, 33^. Recently, in contrast to previous reports stating that rG residues favor anti-conformation in parallel RNA G-quadruplexes, both rG (syn) and rG (anti) have been observed in RNA crystal structures^21^. Additionally, many works have reported the existence of C2′-endo sugars in RNA G-quadruplexes^8, 24, 25, 34^. These observations motivated us to investigate whether an antiparallel RNA G-quadruplex could form in a special sequence context.

The chemical and biochemical properties of G-quadruplexes could be better understood by suitably modifying nucleobases in G-quadruplexes^35, 36^. Galeone *et al*. showed that the substitution of 2′-deoxyguanosine (dG) with 8-methyl-2′-deoxyguanosine (8^m^dG) affects the folding topology of DNA G-quadruplex structures^37, 38^. We previously substituted the dG with 8-bromo-2′-deoxyguanosine (8^Br^dG) in the sequence of human telomere DNA and examined the resultant structures and thermal stabilities, revealing a new mixed-parallel/antiparallel DNA G-quadruplex structure^13, 39^. Similarly, using the 8^m^dG, we also determined the arrangement of the anti/syn conformations of the G-quadruplex structure at the 5′ end of the Rb gene^40^. The incorporation of a methyl or bromine group at the C8 position of dG causes a steric hindrance between the 8-substituent and the ribose ring that favours a syn conformation of 8^m^dG or 8^Br^dG. This allows the examination of the structure and thermal stability of G-quadruplexes via appropriate substitutions of dG with 8^m^dG or 8^Br^dG.

Here, we substituted the rG in the sequence of human telomere 6 nt RNA with 8-bromoguanosine (8^Br^rG) and examined the resultant structures by using NMR, CD, LC-ESI-MS, and gel electrophoresis. For the first time, we successfully observed a new antiparallel RNA G-quadruplex formed by the modified RNA sequence, and further found that two of these G-quadruplexes as subunits, could stack on top of each other and form a dimeric RNA G-quadruplex. Multimethod approaches, CD, NMR, LC-ESI-MS, and gel electrophoresis, have been used to provide several complementary lines of evidence for the existence of antiparallel RNA G-quadruplexes. The availability of the antiparallel RNA G-quadruplex structure now allows us to reconsider the nature of RNA G-quadruplexes, and suggest that RNA may be more polymorphic than initially assumed. These results provide valuable information to allow further understanding of the structural features of RNA G-quadruplexes.

## Results

### CD Studies on 6 nt RNA Sequence

**Figure 1:**
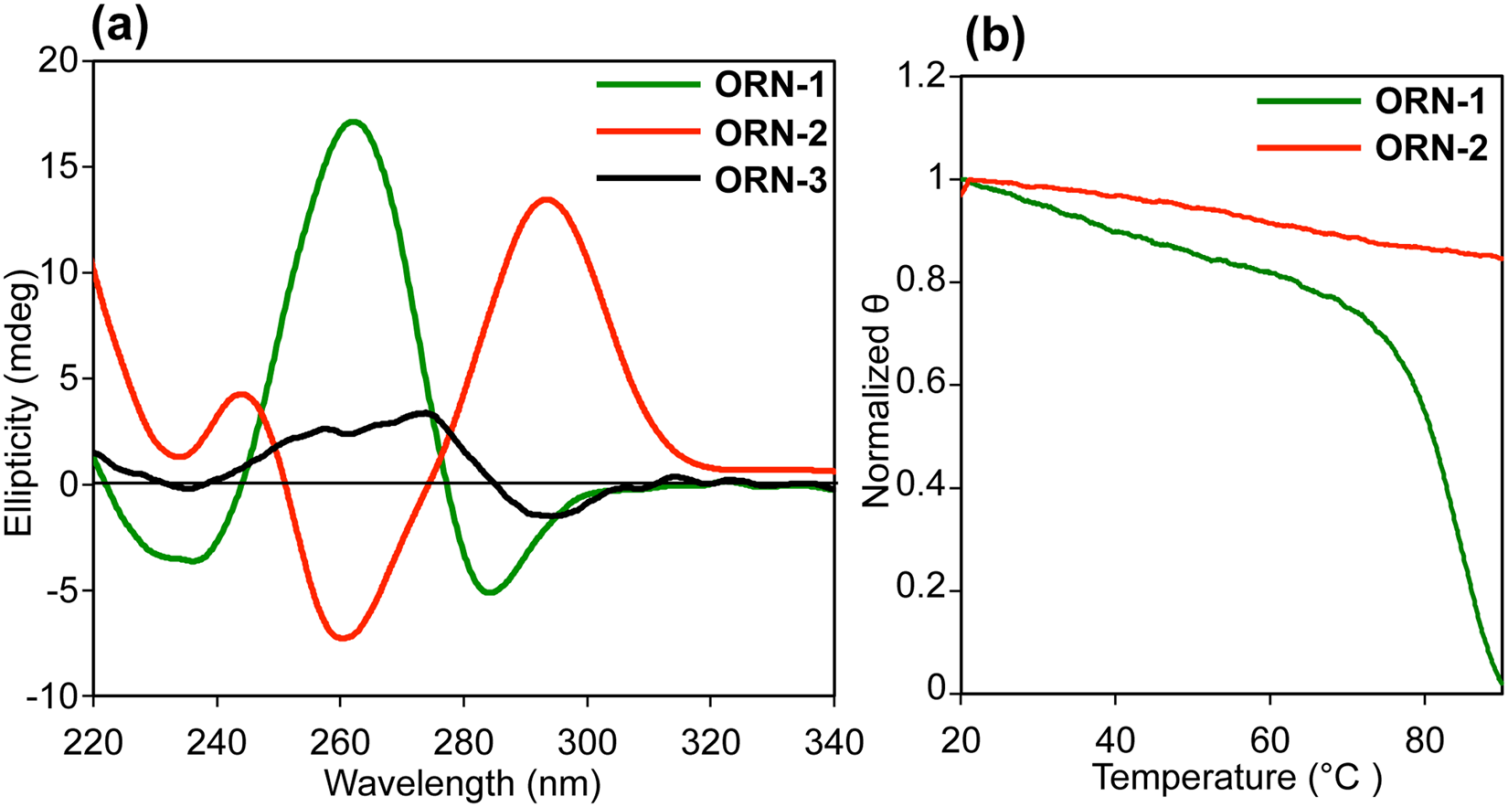
(**a**) CD spectra of ORN-1, ORN-2 and ORN-3 in the presence of 100 mM KCl at 25 °C. (**b**) CD melting curves for ORN-1 and ORN-2 monitored at 265 and 295 nm, respectively.

We substituted three rGs in each of the human telomere RNA sequences r(UAGGGU) with 8^Br^rG to generate ORNs 1–3 (Table 1 and Supplementary Scheme S1 and Figures S1–S8), and examined the resultant structures and their thermal stabilities in a K^+^ solution by CD spectroscopy. The CD spectrum of ORN-1, with a positive band at 265 nm and a negative band at 240 nm, indicated the usual parallel G-quadruplex structure, whereas the CD profile of ORN-3 was characterized as a single strand by the presence of a weak Cotton effect at 275 nm (Fig. 1a). Surprisingly, the ORN-2 showed an unusual CD profile for an RNA G-quadruplex, exhibiting a strong positive band at 295 nm and the negative band at 260 nm, which is typical of an antiparallel G-quadruplex structure (Fig. 1a). The CD spectrum of the unmodified RNA sequence UAGGGU showed a parallel G-quadruplex structure consistent with previously reported (Figure S14)^26^. The thermal stabilities of ORN-1 and 2 were examined using CD melting experiments (Fig. 1b). The Tm value of the parallel G-quadruplex (86.8 °C) suggests that the ORN-1 forms a stable G-quadruplex in presence of 100 mM KCl. Compared with the ORN-1, the melting profile of ORN-2 shows that the antiparallel RNA G-quadruplex is much more stable, with a melting temperature over 90 °C. We further examined the structures and thermal stabilities in a lower KCl concentration. We observed similar CD spectra for ORN-1, ORN-2 and ORN-3 at a concentration of 10 mM (Figure S15). The ORN-2 formed a stable antiparallel G-quadruplex even in the low concentrated K^+^ solution (Figure S15). These data suggest that ORN-2 forms a very stable antiparallel G-quadruplex in K^+^ solution. We also examined the influence of cation on the topology. We found that the antiparallel conformation is formed in sodium but is unable to form in lithium (Figure S16).

**Table 1:** RNA sequences used in this study.

Name	Sequence (5′–3′)
ORN-1	UA(8^Br^rG)GGU
ORN-2	UAG(8^Br^rG)GU
ORN-3	UAGG(8^Br^rG)U

### NMR Spectral Assignment of ORN-2

**Figure 2:**
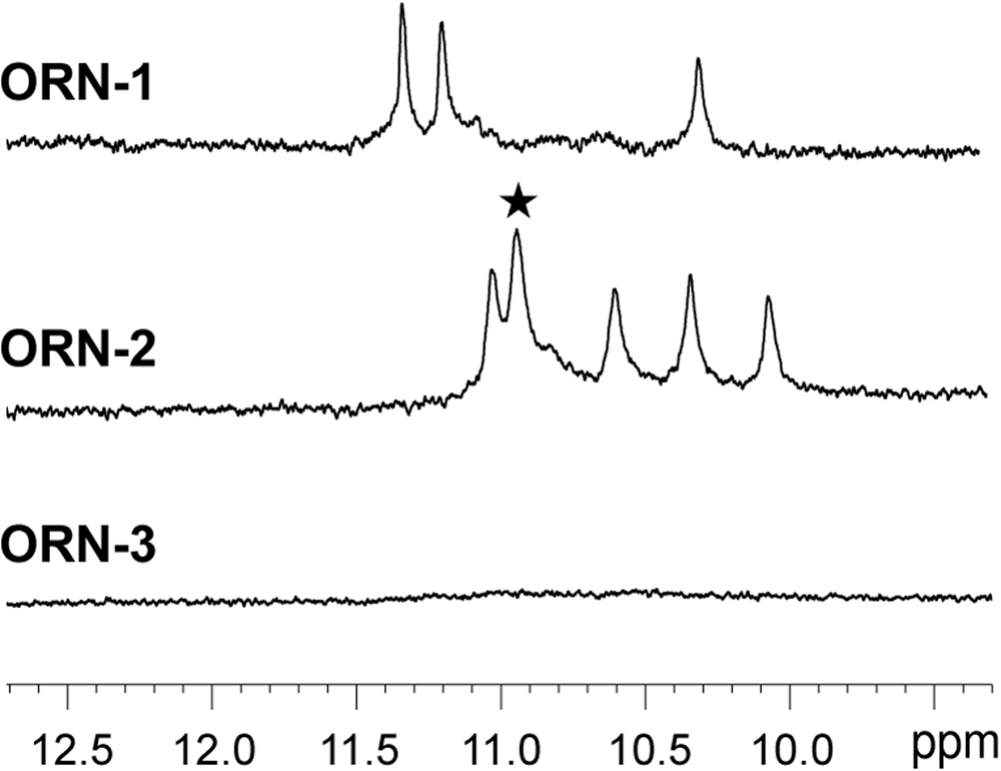
Imino proton NMR spectra of ORN-1, ORN-2 and ORN-3 in the presence of 100 mM KCl and 10 mM potassium phosphate buffer (pH 6.8), 25 °C. Asterisk indicates an overlapping of two peaks.

**Figure 3:**
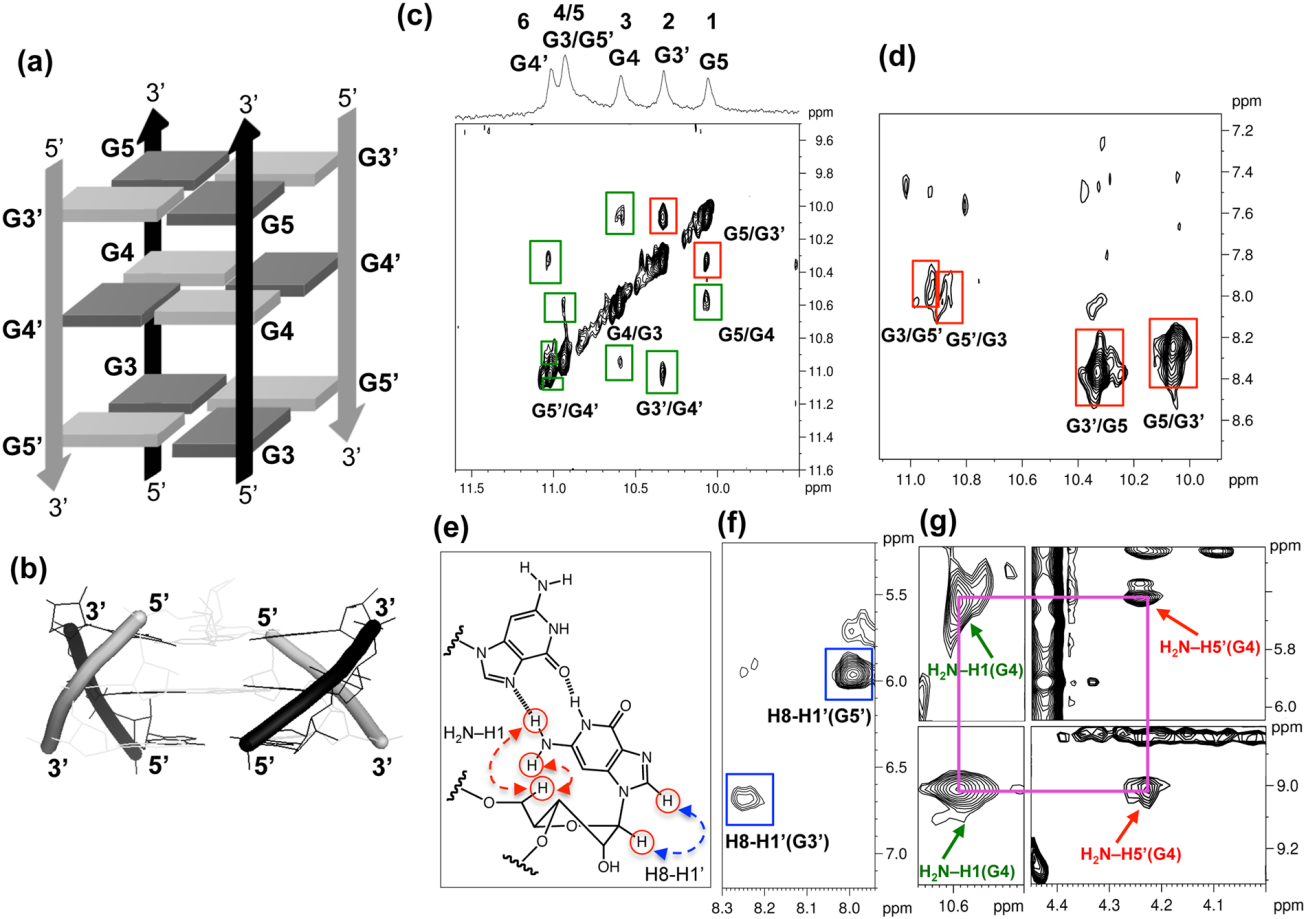
(**a**) Schematic structure of ORN-2, where syn and anti residues are in grey and black, respectively. For clarity, the two unequivalent strands with the contrary orientation are labeled 5′-U1-A2-G3-G4-G5-U6-3′ (black) and 5′-U1′-A2′-G3′-G4′-G5′-U6′-3′ (grey), U and A residues have been omitted. (**b**) Side view of the antiparallel G-quadruplex structure. (**c**) H1-H1 region and (**d**) H1-H8 region of 2D-NOESY spectra of ORN-2 in the presence of 100 mM KCl and 10 mM potassium phosphate buffer; imino peaks were labeled with correspond numbers. Intratetrad and sequential connectivity were represented by red and green boxes, respectively. (**e**) Schematic representation of syn-glycosidic conformation of rG with arrows indicating the H8-H1′ and amino protons-H5′ NOEs. (f) H8-H1′ region of 2D-NOESY spectrum of ORN-2. Intraresidue H8-H1′ cross peaks of G3′ and G5′ indicate that the two rG residues were syn conformation. (**g**) NOEs of G4 are labeled with purple frame. The red allows indicated NOE peaks of non-hydrogen-bonded-and hydrogen-bonded-amino protons of G4 with itself H5′ (upper and lower). The green allows indicated NOE peaks of non-hydrogen-bonded- and hydrogen-bonded-amino protons of G4 with itself H1 (upper and lower).

We further investigated the structure of ORNs 1–3 by NMR. In the imino proton region of the ^1^H NMR spectrum of ORN-1 obtained in the presence of K^+^, three well-defined signals were observed between 10.0 and 11.5 ppm (Fig. 2), which were consistent with the symmetrical parallel G-quadruplex showed by CD spectroscopy. ORN-3 did not show the imino signal, meaning that ORN-3 was not able to form a G-quadruplex, which confirms the CD spectral results. Surprisingly, with ORN-2, we observed five imino peaks with an overlap of two peaks, suggesting that the G-quadruplex structure formed by ORN-2 is far more complex than that of ORN-1, in which a twice higher intensity peak presented by two overlapped peaks (marked by asterisk). Since the strands of the UAGGGU parallel G-quadruplex are equivalent, three G-quartet rings, themselves formed by twelve G residues, should display three imino protons, as for ORN-1. The presence of these six imino peaks suggested that the strands of the G-quadruplex were not equivalent in terms of orientation, and folding in an antiparallel to yield unequivalent G residues (Fig. 3a,b). For antiparallel G-quadruplex, there are two possible patterns of G-stem orientation, “ppaa” and “papa” model structures (Figure S9).

We further performed NMR spectral assignment by a combination of hydrogen-deuterium exchange (HDX), homonuclear NOESY, and heteronuclear [^13^C-^1^H] HMBC experiments at natural abundance^24, 41, 42^. The imino protons assignments were based on the through-bond correlation between H8 and imino protons via ^13^C5 at natural abundance of each individual rG (Figure S10). Due to the missing H8 of the two residues (H is replaced by Br), the imino peaks (peak 3 and 5) that do not have the NOEs with the H8 can be assigned as G4 or G4′ (Fig. 3c). Both of peak 1 and peak 4 have NOEs with G4, suggesting the G residues from peak 1 and 4 are in the same strand with the G4. We can conclude that the two peaks are G3 and G5, with the same reason the peak 2 and 5 are assigned to G3′ and G5′. To accurately detect the peak 1, we assigned the peaks by the assistant of H8/H6-H1′ sequential connectivity (Figure S11). Through the H8/H6-H1′ sequential connectivity traced in the NOESY spectrum of Figure S11, the H8-H1′ NOEs signal of G3′ could be found. Through the intratetrad cyclic NOEs connectivity (between H1 and H8), we determined the H1 of G5 and assigned the peak1 as G5. The peaks 2, 4 and 5 are further assigned using the same sequential connectivity method, respectively.

To further understand the structure feature, we assigned the NOE connectivities in detail. The intratetrad NOE connections G5H1/G3′H1 (GH1/GH1) and G5H1/G3′H8, G3′H8/G5H1 (GH1/GH8) revealed the G tetrad formation of G3′-G5-G3′-G5 (Fig. 3c,d). The G tetrad G3-G5′-G3-G5′ was also defined by G5′H1/G3H8 and G3H1/G5′H8 (GH1/GH8) (Fig. 3d). The sequential NOE interactions G4H1/G3H1, G5H1/G4H1, G3′H1/G4′H1, and G5′H1/G4′H1 correspond to imino protons of G on adjacent G-tetrads for G3-G4-G5 and G3′-G4′-G5′ steps on each strand (Fig. 3c). The chain from the 5′ to 3′ end seen by monitoring the H8/H6-H1′ sequential connectivity of ORN-2 could be traced in the NOESY spectra, while the connectivities were interrupted at the 8^Br^rG positions because of the lack of H8 of G4′ (Figure S11).

The syn-glycosidic conformations of G3′ and G5′ for the antiparallel structure is supported by the observation of the strong presence of intraresidue H8/H1′ NOEs (Fig. 3e,f). Due to the lack of H8 for G4 to detect those conformations, we used amino protons to identify glycosidic conformations. We observed NOE connectivities of both non-hydrogen-bonded and hydrogen-bonded-amino protons of G4 with itself H5′ (Fig. 3e,g), indicating that it forms a syn conformation.

**Figure 4:**
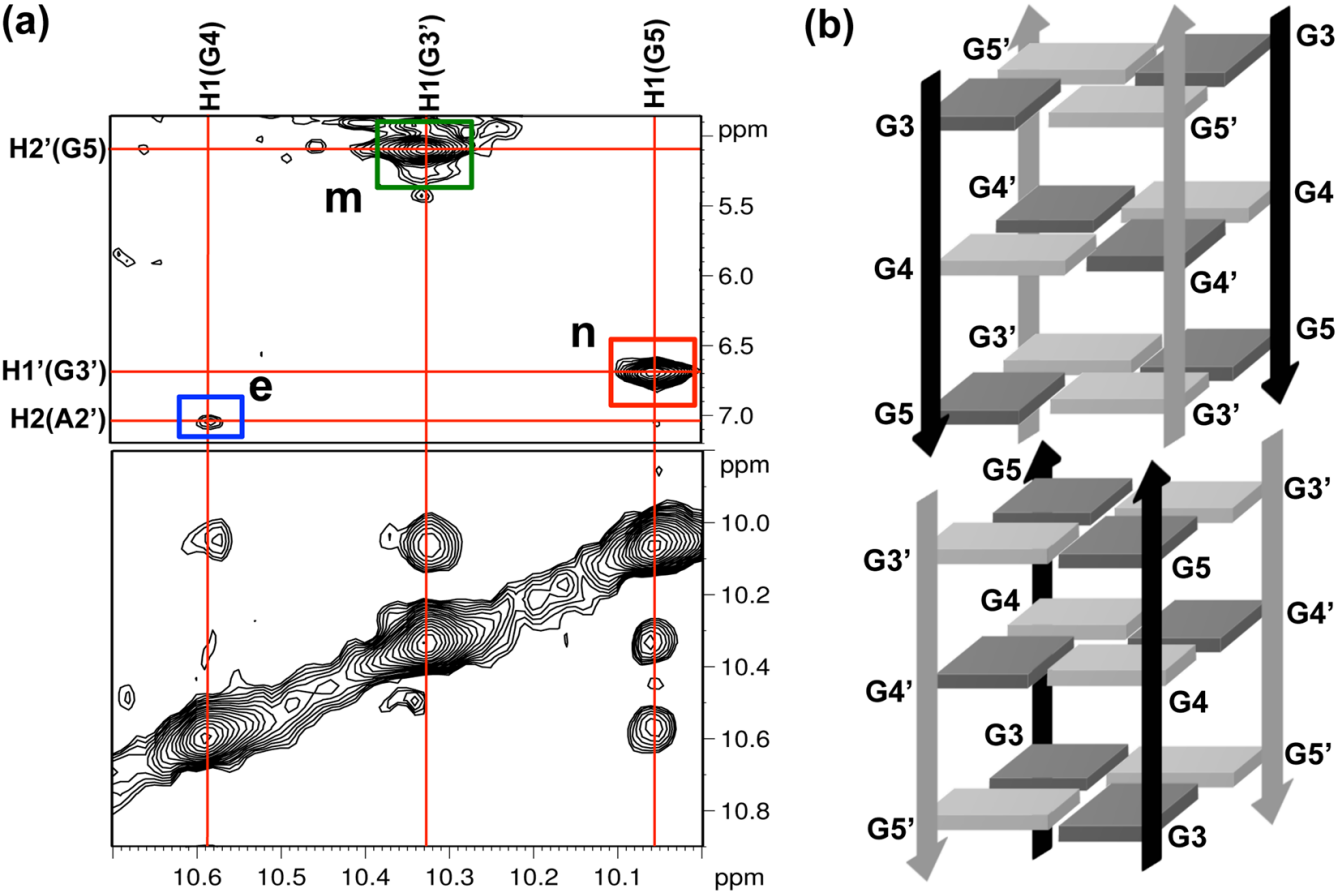
(**a**) NOE peaks of the ORN-2 indicating the stacking between two G-quadruplexes are framed and labeled: n, H1(G5)–H1′(G3′); m, H1(G3′)–H2′(G5); e, H1(G4)–H2(A2′). (**b**) Schematic structure of dimer RNA G-quadruplex.

Interestingly, we observed NOE peaks between G5 and G3′ (G5H1-G3′H1′, G3′H1-G5H2′) (Fig. 4a), which can only be explained by the fact that the ORN-2 forms a dimeric G-quadruplex and that the stacking effect brings the G3′, present in one subunit G-quadruplex, close enough to the G5 of the next subunit (Fig. 4b). The cross-peak between G4 imino and the A2′H2 was also observed (Fig. 4a), implying a fact that the A from UA at 5′-teminal of one subunit G-quadruplex was close to the G4-G4′-G4-G4′ tetrad of another subunit G-quadruplex (Fig. 4b). These results further confirmed the dimeric G-quadruplex structure formation. The two such structures are sandwiched on both sides of the two G-tetrads of the respective G-quadruplex subunit. These novel structural features, involving intensive stacking of adjacent G-G-G-G tetrads, should contribute to the stability of the dimer G-quadruplex.

That result is also supported by hydrogen-deuterium exchange (HDX) experiment. G3′ and G5 at the G3′-G5-G3′-G5 tetrad from the inside of dimer structure were more resistant to the solvent exchange in comparison with G3 and G5′ at the G3-G5′-G3-G5′ tetrad from the outside of dimer structure (Figure S12), suggesting that G residues on stacking top were not easily exchanged due to the protecting effect of tetrad stacking on the inside of structure. In addition, the H1-H8 signals of G3′ and G5 at the G3′-G5-G3′-G5 tetrad showed a higher intensity than that of G3 and G5′ at the G3-G5′-G3-G5′ (Fig. 3d), suggesting that the stacking effect makes G3′ and G5 more close to each other at the tetrad, whereas due to the Br modification, G4 and G4′ do not have the H8 that leads to the absence of H1-H8 signal.

Furthermore, taking into account the presence of 5′-UA and U-3′ flanking nucleotides that might form an additional platform (U-U-U-U or U-A-U-A tetrad) to stabilize the structure, we checked the imino region around 13–14 ppm to find whether U-A Watson-Crick pairs form U-A-U-A tetrad and did not observed the peaks around the region (Figure S13), indicating that UAUA platform is not constructed from the 5′-UA. We also did not find the NOE peaks of H3-H5, H3-H6 from the U-3′, suggesting that there is no U-tetrad formed by the U-3′ in the structure.

### LC-ESI-MS and Gel Electrophoresis studies on ORN-2

**Figure 5:**
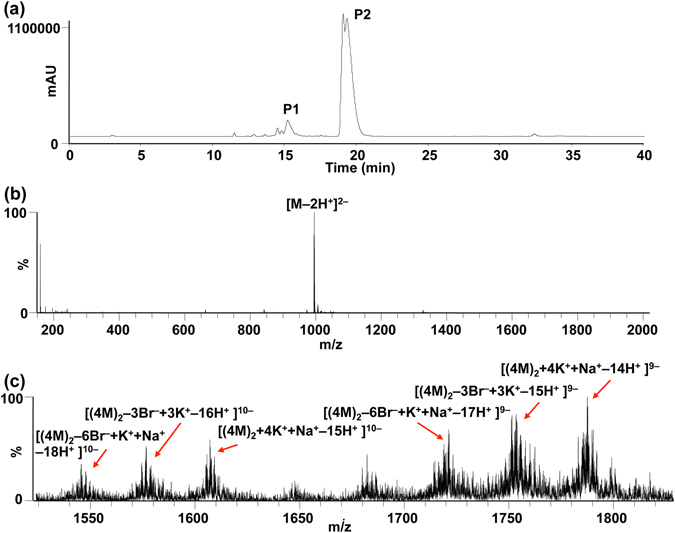
(**a**) LC analysis result of ORN-2. UV absorbance is monitored at 254 nm. (**b**) ESI-MS spectrum of P1 peak in LC. (**c**) ESI-MS spectrum of P2 peak in LC. Molecular ions (9^–^ and 10^–^) are directly observed for dimer G-quadruplex by electrospray ionization MS. Sample concentration is 0.05 mM. M: molecular weight.

Several methods such as LC-ESI-MS^28, 43^ and DOSY^44, 45^ have been used for determining the molecular weight of G-quadruplex. Next, we performed the LC-ESI-MS experiment to further analyze the ORN-2. As shown in Fig. 5a, the two peaks (P1 and P2) in LC spectrum were observed with retention times of 15.5 and 19.3 min, respectively. The minor peak P1 corresponds to the molecular weight (MW = 1992.1) of ORN-2, found in the MS spectrum (Fig. 5b). The associated molecular weight for the major peak P2 is consistent with a dimeric G-quadruplex of ORN-2 (of mass 4 MW × 2), in which the ions near m/z [1788.5] and [1609.2] were interpreted as [(4 M)_2_ + 4 K^+^  + Na^+^ − 14 H^+^]^9−^ and [(4 M)_2_ + 4 K^+^  + Na^+^ − 15 H^+^]^10–^ (Fig. 5c). Additionally, we also observed the different fragmentation patterns of the dimer G-quadruplex in the MS spectrum, in which the Br^−^ loss as a primary fragmentation pathway produced the [(4 M)_2_−3Br^–^ + 3 K^+^ − 15 H^+^]^9−^, [(4 M)_2_ − 6Br^−^ + K^+^  + Na^+^ − 17 H^+^]^9−^, [(4 M)_2_ − 3Br^−^ + 3 K^+^ − 16 H^+^]^10−^, and [(4 M)_2_ − 6Br^−^ + K^+^  + Na^+^ − 18 H^+^]^10−^ peaks (Fig. 5c)^43^. Therefore, LC-ESI-MS supports that two G-quadruplexes stack each other to form a dimeric RNA quadruplex. We performed the LC-MS experiments to compare between the unmodified, ORN-1, 2 and 3 sequences. We observed the LC-MS peaks of UAGGGU and ORN-1 corresponding to both the tetramer and monomer. Only monomer peak was found in ORN-3 (Figure S17). These observations are consistent with the NMR and CD results.

**Figure 6:**
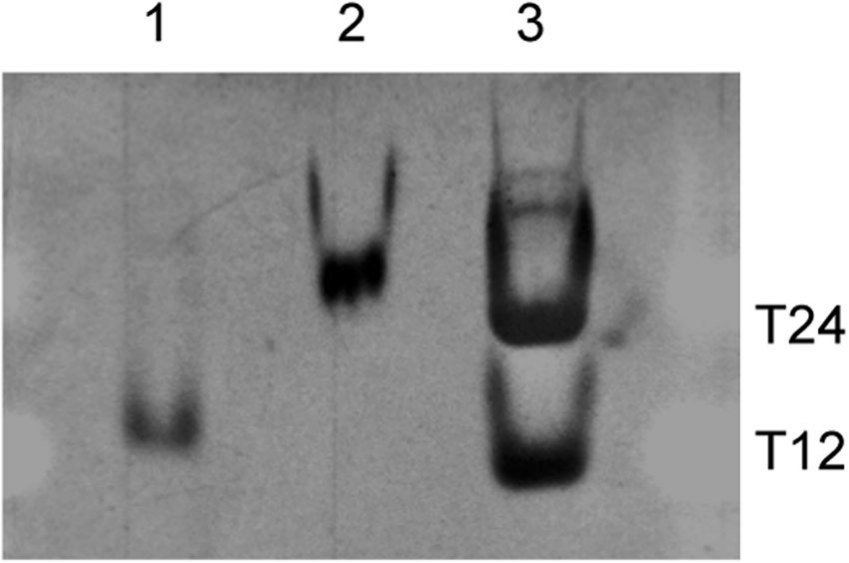
Nondenaturing gel electrophoresis of ORN-2. Lane 1: native 6 nt r(UAGGGU) as a control, lane 2: ORN-2, lane 3: markers dT12 and dT24.

Furthermore, we carried out non-denaturing gel electrophoresis to investigate the structure of ORN-2. Contrary to the control RNA r(UAGGGU), the ORN-2 showed a reduced mobility compared with the reference DNA oligonucleotide dT24 (Fig. 6), indicating a higher order G-quadruplex formation.

## Conclusions

Although there is no report that confirmed any RNA sequences can form an antiparallel G-quadruplex conformation, recently, accumulated evidence showed that RNA G-quadruplexes present some antiparallel features (such as the complex loops and the antiparallel characteristic CD signature), suggesting that the RNA maybe more polymorphic than initially assumed. For example, Gabelica and coauthors have reported that the relative intensity of CD band at 295 nm was observed for some RNA sequences, suggestive of a mixed arrangement of the guanine steps in RNA G-quadruplexes^27^. The crystal structure study of RNA aptamer (PDB code: 3IVK) revealed the complex loops of G-quadruplex structure, which were described as “non-parallel”^21^. To find the typical antiparallel RNA G-quadruplex with different orientations in G-stem, Mergny *et al*. applied the hybrid duplex–quadruplex structure and tried to induce the formation of a RNA antiparallel orientation in G-stem^22^. Furthermore, Defrancq *et al*. applied a template strategy for constraining RNA to adopt an antiparallel topology, they were unable to form antiparallel RNA^46^. The DNA G-quadruplex was successfully transformed into an antiparallel conformation, unlike the RNA G-quadruplex that was found difficult to form an antiparallel orientation in G-stem. The results suggested that the G-stem structure itself, and not the loops, maybe the key element when it comes to forcing RNA G-quadruplexes into a parallel conformation. Accordingly, the syn/anti-glycosidic conformation of rGs is an important factor in the G-stem orientation of RNA G-quadruplexes^47^. Consequently, stabilizing the syn-glycosidic conformation of rGs in G-stem can drive the formation of antiparallel G-quadruplexes.

In this study, by using the 8-substituted purine 8^Br^rG to stabilize the syn conformation, we found that the modified ORN-2 RNA sequence was able to form an antiparallel RNA G-quadruplex, which had never been reported before, and further observed a dimeric RNA G-quadruplex, adopted by stacking two of these G-quadruplexes. Interestingly, the replacement of the central rG in the G-stem results in the formation of an antiparallel G-quadruplex (ORN-2), while the replacement of the first rG in the sequence does not affect the parallel (ORN-1) and the third rG substitution does not induce any folding (ORN-3), suggesting that the rG at different positions of G-stem exerted differential effects on G-quadruplex folding. A recent report also suggested the similar position-dependent effect^48^. The G at central position has more effect on G-quadruplex structure than the G at the other positions. Antiparallel RNA G-quadruplex formation provides new insights into the RNA structure and leads to a better understanding of the essential biological role of RNA molecules.

## Methods

### RNA Synthesis and Purification

The syntheses of the 8-bromoguanosine-containing RNAs were carried out by phosphoramidite chemistry. All RNAs were synthesized on the 1 μmol scale with an automatic DNA/RNA synthesizer (Nihon Techno Service Co., LTD.). After automated synthesis, the oligonucleotides were detached from the support and deprotected according to the manufacturer’s protocol. All oligonucleotides were purified by Reverse phase-HPLC (JASCO). All synthetic procedures and compound characterizations are described in the Supporting Information. RNA sequences are Table 1.

### CD Measurements and Analysis of CD Melting Profile

CD spectra were measured using a Jasco model J-810 CD spectrophotometer. Samples were prepared by heating the oligonucleotides at 90 °C for 5 min and gradually cooling them to room temperature. The melting curves were obtained by monitoring a 265 and 295 nm CD band. Solutions for CD spectra were prepared as 0.3 mL samples at 0.01 and 0.1 mM concentrations in the presence of 10 and 100 mM KCl or in the absence of KCl, 10 mM potassium phosphate buffer (pH 6.8).

### NMR Experiments

NMR experiments were performed a BRUKER (AV-400M) magnetic resonance spectrometer. Spectra were recorded at 25 °C. RNA samples (0.6–5.2 mM) were dissolved in 0.15 mL of 90% H_2_O/10% D_2_O, 10 mM potassium phosphate, pH 6.8, 100 mM KCl. Assignments of the proton resonances were initially made by using the methods previously described for assignments of G-quadruplex RNA structures. These assignments were confirmed by comparison of NOESY spectra to those of the related telomere RNA^20, 23, 26, 49, 50^. HDX Experiments: RNA samples (5.2 mM in 0.15 mL H_2_O) were lyophilized overnight prior to the HDX experiment. 0.15 mL D_2_O was added to resolve the sample just before measurement. Three samples were prepared, and the ^1^H NMR spectra were recorded, respectively. For each sample, the spectrum was recorded after the specific D_2_O exchange time, measurement time is 10 min.

### LC-ESI-MS

LC-ESI-MS experiments were performed on Q Exactive Quadrupole-Orbitrap LC-MS System (Thermo Scientific) equipped with a XBridge Oligonucleotide Separation Technology C18 column (2.5 µm, 4.6 mm × 50 mm, Waters). Data was acquired using Xcalibur software. Sample was prepared at 0.05 mM concentration in 100 mM KCl. LC condition: A solution (H_2_O with 50 mM triethylammonium acetate); B solution (H_2_O: MeOH = 1: 1 with 50 mM triethylammonium acetate); flow rate was 0.2 ml/min. A spray voltage 3.5 kV was used to generate the negative ion mode with a heated capillary temperature of 250 °C. An ion accumulation time was 100 ms.

### Gel Electrophoresis

Nondenaturing gel electrophoresis experiments were performed in a 0.5 × TBE buffer in the presence of 20 and 100 mM KCl. Electrophoresis experiments were run at 80 V for 5 h at 4 °C, and the gel was viewed by GelStar (Lonza) staining. RNA samples concentration ranged from 90 to 140 pmol. DNA oligonucleotides dT12 and dT24 were used as molecular markers.

## Electronic supplementary material

Supplementary information
