# Supplementary material for: Antiparallel RNA G-quadruplex Formed by Human Telomere RNA Containing 8-Bromoguanosine
Source: Sci Rep. 2017 Jul 27;7:6695. doi: 10.1038/s41598-017-07050-w (PMC5532209; doi:10.1038/s41598-017-07050-w)
Supplement: Supplementary file 1 — Supplementary information [file 41598_2017_7050_MOESM1_ESM.pdf]

Supporting Information

Antiparallel RNA G-quadruplex formed by human telomere RNA containing 8-bromoguanosine

Chao-Da Xiao, Takumi Ishizuka, Yan Xu*

Division of Chemistry, Department of Medical Sciences, Faculty of Medicine, University of Miyazaki, Japan.

General.

^{1}H-NMR and ^{31}P-NMR spectra were recorded on a BRUKER (AV-400M) magnetic resonance spectrometer. DMSO-d_6 and $CDCl_3$ were used as the solvents. ^{1}H spectra chemical shifts (δ) are reported in parts per million (ppm) referenced to residual protonated solvent peak (DMSO-d_6, δ = 2.50, $CDCl_3$, δ = 7.26). Coupling constants (J) values are given in hertz (Hz). Signal patterns are indicated as br (broad), s (singlet), d (doublet), t (triplet), sept (septet), m (multiplet). All reagents were purchased from Aldrich, TCI (Tokyo Chemical Industry) or Wako (Wako Pure Chemical Industries). Thin layer chromatography (TLC) was performed using TLC Silica gel 60 F_{254} (Merck). Compounds were visualized using a UV lamp (254 nm) or staining with a potassium permanganate solution. Silica gel (Wakogel® C-300, 200-325 mesh) was used for column chromatography. Purification of products was also performed on a middle pressure liquid chromatography (MPLC) systems (EPCLC-AI-580S, Yamazen Corporation) equipped with silica gel column (Hi-Flash Column, Yamazen Corporation) and Recycling Preparative HPLC system (LC-9201, Japan Analytical Industry) with JAIGEL-1H and 2H columns with $CHCl_3$ as an eluent running at 3.5 mL/min. High-resolution mass spectra (HRMS) were recorded by electrospray ionization (ESI) on an Exactive Orbitrap mass spectrometer instrument (Thermo Scientific).

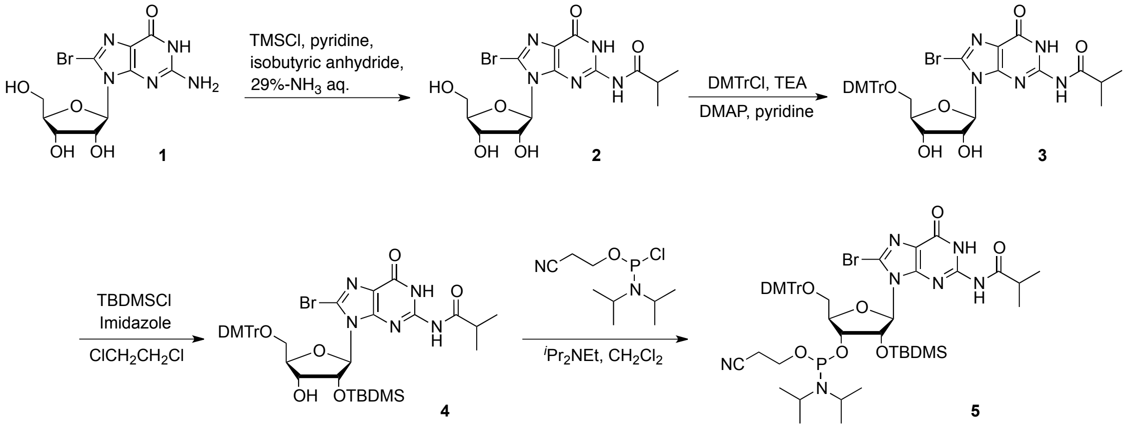

Scheme S1 Synthetic scheme of 8-bromoguanosine phosphoramadite **5**.

The synthesis of 8-bromoguanosine phosphoramadite **5** was modified from procedures described by Proctor *et al.*[1] 8-Bromoguanosine **1** was purchased from TCI (B4002, Tokyo Chemical Industry)

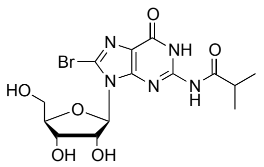

***N*^{2}-Isobutyryl-8-bromoguanosine (2)** 8-Bromoguanosine **1** (10 g, 27.8 mmol) was co-evaporated with pyridine (40 mL) three times, followed by suspension in anhydrous pyridine (40 mL), and trimethylchlorosilane (19.5 mL, 139 mmol) was added. The mixture was stirred for 2 h at room temperature, isobutyric anhydride (21 mL, 125 mmol) was added, and the mixture was stirred for 4 h at room temperature under argon. The reaction was cooled in an ice bath, and 35 mL of water was added. After 15 min, 35 mL of 29 % aqueous ammonia was added, and the reaction was stirred for 15 min. The solution was then evaporated to near dryness, and the residue was dissolved in 500 mL of water. The mixture was extracted twice with 200 mL of dichloromethane. The organic layer was dried over Na_2SO_4, and concentrated *in vacuo*. The target compound was given by silica gel column chromatography: 8.1 g, yield was 68 %. ^{1}H-NMR (400 MHz, DMSO-d_6) δ 12.20 (s, 1H), 11.54 (s, 1H), 5.80 (d, J = 6.2 Hz, 1H), 5.53 (d, J = 5.4 Hz, 1H), 5.14 (d, J = 4.5 Hz, 1H), 5.06 (q, J = 5.2 Hz, 1H), 4.80 (t, J = 5.6 Hz, 1H), 4.19 (m, 1H), 3.85 (dt, J = 9.8, 3.9 Hz, 1H), 3.67 (m, 1H), 3.52 (m, 1H), 2.79 (sept, J = 6.8 Hz, 1H), 1.13 (d, J = 6.8 Hz, 6H). HRMS (ESI) for $C_{14}H_{17}O_6N_5Br$ $[M-H]^-$: Calcd. 430.0357; Found. 430.0366.

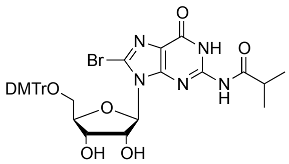

***N*^{2}-Isobutyryl-5'-*O*-dimethoxytrityl-8-bromoguanosine (3)** The compound **2** (6.4 g, 14.8 mmol) was co-evaporated with pyridine (30 mL) three times and suspended in 25 mL dry pyridine. 4,4'-Dimethoxytrityl chloride (9 g, 47.6 mmol), triethylamine (3.84 mL, 26.9 mmol), and 4-dimethylaminopyridine (54 mg, 0.51 mmol) were added to the mixture. After it stood overnight, TLC (CH_2Cl_2 : CH_3OH = 10 : 1) showed complete reaction. The reaction was cooled in an ice bath, and 50 mL of aqueous 5 % $NaHCO_3$ solution was added. The mixture was extracted twice with 60 mL of dichloromethane. The organic layer was dried over Na_2SO_4, and concentrated *in vacuo*. The target compound was given by silica gel column chromatography: 6.7 g. Yield was 62 %. ^{1}H-NMR (400 MHz, DMSO-d_6) δ 12.14 (s, 1H), 11.36 (s, 1H), 7.31-7.28 (m, 2H), 7.18-7.14 (m, 7H), 6.76 (d, J = 7.0 Hz, 2H), 6.70 (d, J = 7.0 Hz, 2H), 5.87 (d, J = 4.6 Hz, 1H), 5.66 (d, J = 5.8 Hz, 1H), 5.08 (d, J = 6.6 Hz, 1H), 5.01 (q, J = 5.0 Hz, 1H), 4.32 (q, J = 6.0 Hz, 1H), 4.05 (m, 1H), 3.71 (s, 3H), 3.69 (s, 3H), 3.42 (dd, J = 10.3, 7.8 Hz, 1H), 3.11 (dd, J = 10.3, 2.5 Hz, 1H), 2.72 (sept, J = 6.8 Hz, 1H), 1.12 (d, J = 6.5 Hz, 3H), 1.10 (d, J = 6.6 Hz, 3H). HRMS (ESI) for $C_{35}H_{35}O_8N_5Br$ $[M-H]^-$: Calcd. 732.1664; Found. 732.1675.

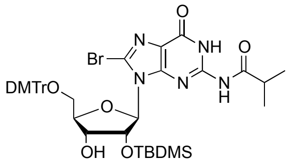

***N*^{2}-Isobutyryl-2'-*O*-(*tert*-butyldimethylsilyl)-5'-*O*-dimethoxytrityl-8-bromo guanosine (4)** The compound **3** (4.0 g, 5.4 mmol) was co-evaporated with anhydrous pyridine (30 mL) three times and dissolved in 30 mL of anhydrous pyridine. Then, imidazole (0.9 g, 13.7 mmol) and *tert*-butyldimethylsilyl chloride (1.0 g, 6.8 mmol) were added and left for 1 h at room temperature. The reaction mixture was worked up with a saturated $NaHCO_3$ solution (50 mL), and the mixture was extracted three times (50 mL) with dichloromethane. The organic layer was dried over Na_2SO_4, and concentrated *in vacuo*. The target compound was given by silica gel column chromatography (*n*-hexane : ethyl acetate = 97 : 3): 1.6 g. Yield was 35 %. The TBDMS group that protected 2' hydroxyl group in the compound 4 was conformed by ^{1}H-^{1}H COSY experiment, where clear cross peaks were observed between the 3'H and 3'OH (See Figure S6). ^{1}H-NMR (400 MHz, DMSO-d_6) δ 12.15 (s, 1H), 11.31 (s, 1H), 7.34-7.31 (m, 2H), 7.21-7.14 (m, 7H), 6.79 (d, J = 8.9 Hz, 2H), 6.74 (d, J = 8.8 Hz, 2H), 5.88 (d, J = 4.8 Hz, 1H), 4.91 (m, 2H), 4.30 (m, 1H), 4.08 (m, 1H), 3.71 (s, 3H), 3.67 (s, 3H), 3.46 (dd, J = 10.2, 7.6 Hz, 1H), 3.16 (dd, J = 10.2, 3.2 Hz, 1H), 2.68 (sept, J = 6.8 Hz, 1H), 1.10 (d, J = 6.8 Hz, 3H), 1.08 (d, J = 6.9 Hz, 3H), 0.78 (s, 9H), -0.02 (s, 3H), -0.12 (s, 3H). HRMS (ESI) for $C_{41}H_{49}O_8N_5BrSi$ $[M-H]^-$: Calcd. 846.2528; Found. 846.2538.

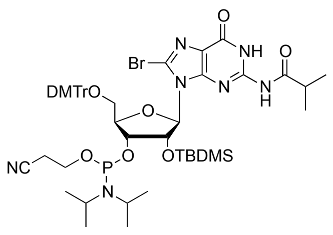

***N*^{2}-Isobutyryl-2'-*O*-(*tert*-butyldimethylsilyl)-5'-*O*-dimethoxytrityl-8-bromoguanosine phosphoramidite (5)** The compound **3** (1.6 g, 1.7 mmol) was co-evaporated with anhydrous acetonitrile (20 mL) and anhydrous dichloromethane (4 mL) three times, followed by suspension in anhydrous acetonitrile (20 mL). Next, *N*,*N*-diisopropylethylamine (1.2 mL, 7.6 mmol) and 2-cyanoethyldiisopropylchlorophosphoramidite (1 mL, 5.7 mmol) were added and left for 2 h at room temperature. The reaction mixture was worked up with an aqueous 5 % $NaHCO_3$ solution (20 mL), and the mixture was extracted three times (20 mL) with dichloromethane containing 1% triethylamine. The organic layer was dried over Na_2SO_4, and concentrated *in vacuo*. The reaction mixture was purified by a middle pressure liquid chromatography (MPLC) in *n*-hexane containing 1 % triethylamine with a gradient formed by ethyl acetate: 1.5 g. Yield was 76 %. ^{1}H-NMR (400 MHz, DMSO-d_6) δ 11.96 (s, 1H), 7.62 (m, 2H), 7.53-7.43 (m, 4H), 7.25 (m, 3H), 6.83-6.76 (m, 1H), 6.01 (d, J = 8.3 Hz, 2H), 5.48 (dd, J = 8.1, 5.7 Hz, 1H), 4.39-4.32 (m, 1H), 4.22 (m, 1H), 4.05-3.98 (m, 1H), 3.96-3.87 (m, 1H), 3.77 (s, 3H), 3.75 (s, 3H), 3.68-3.43 (m, 5H), 2.99-2.90 (m, 1H), 2.76-2.63 (m, 1H), 1.14 (d, J = 6.7 Hz, 6H), 0.90 (d, J = 6.8 Hz, 6H), 0.82 (s, 9H), 0.70 (d, J = 6.7 Hz, 3H), 0.39 (d, J = 6.6 Hz, 3H), 0.04 (s, 3H), -0.15 (s, 3H). ^{31}P-NMR (161 MHz, $CDCl_3$) δ 151.67, 148.6. HRMS (ESI) for $C_{50}H_{66}O_9N_7BrPSi$ $[M-H]^-$: Calcd. 1046.3607; Found. 1046.3613.

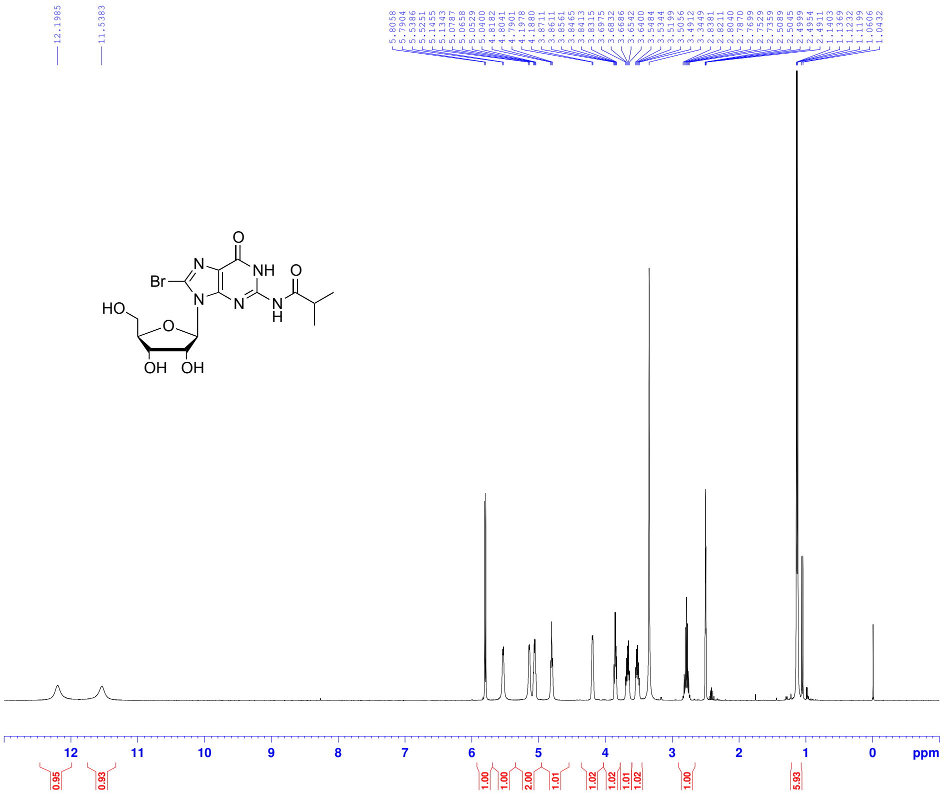

Figure S1. ^{1}H NMR spectrum of compound **2**.

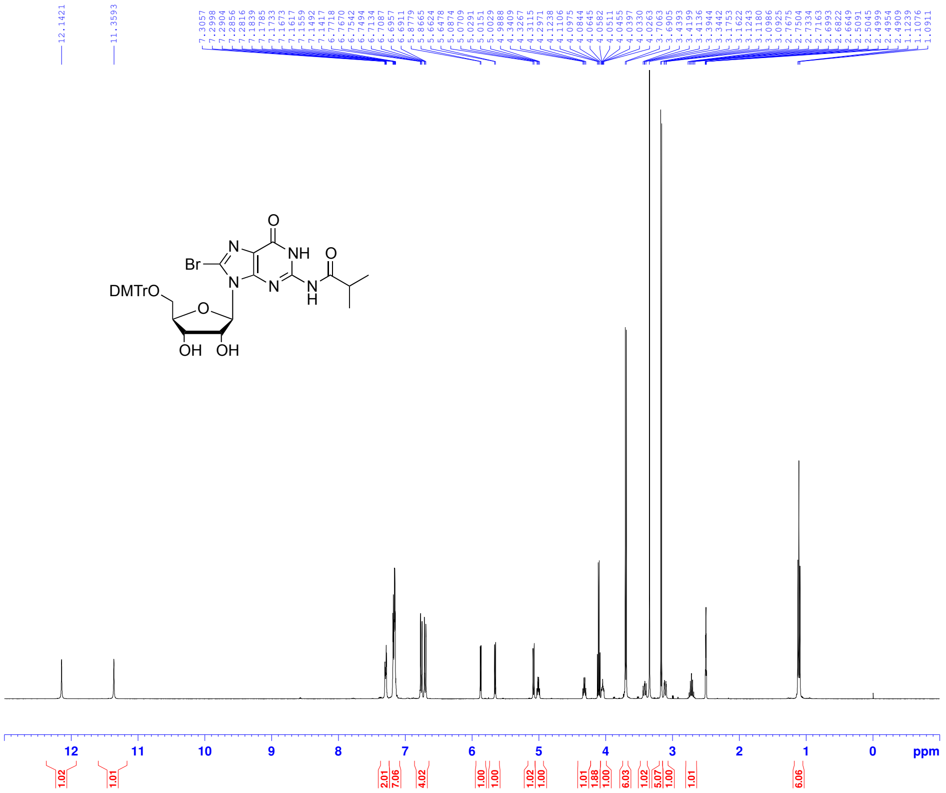

Figure S2. ^{1}H NMR spectrum of compound **3**.

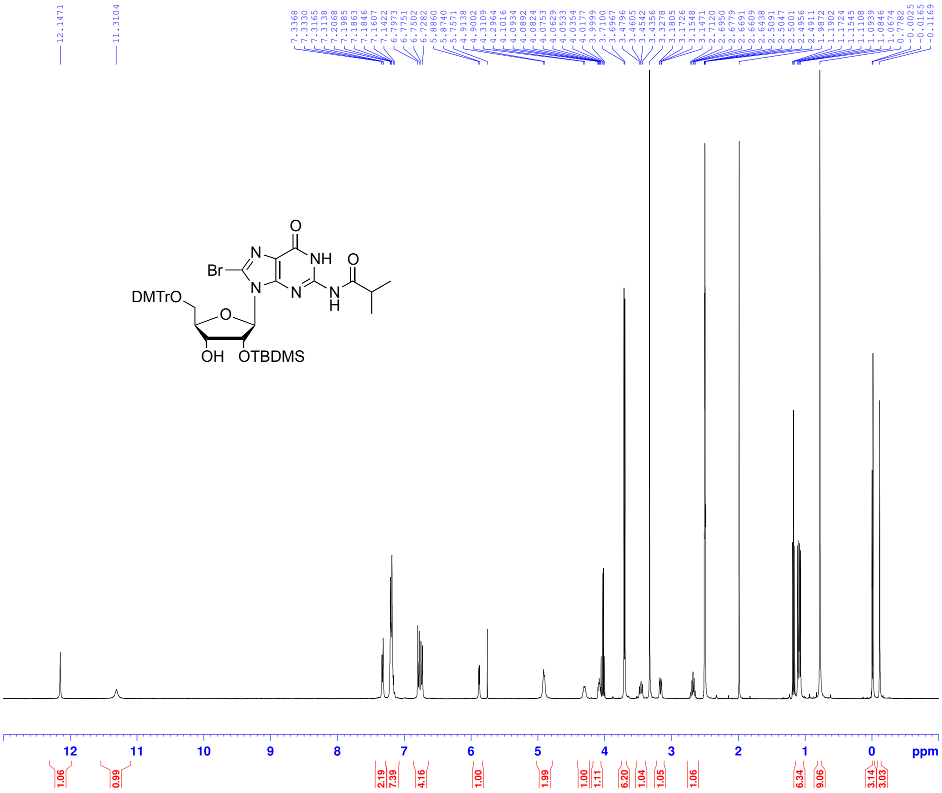

Figure S3. ^{1}H NMR spectrum of compound **4**.

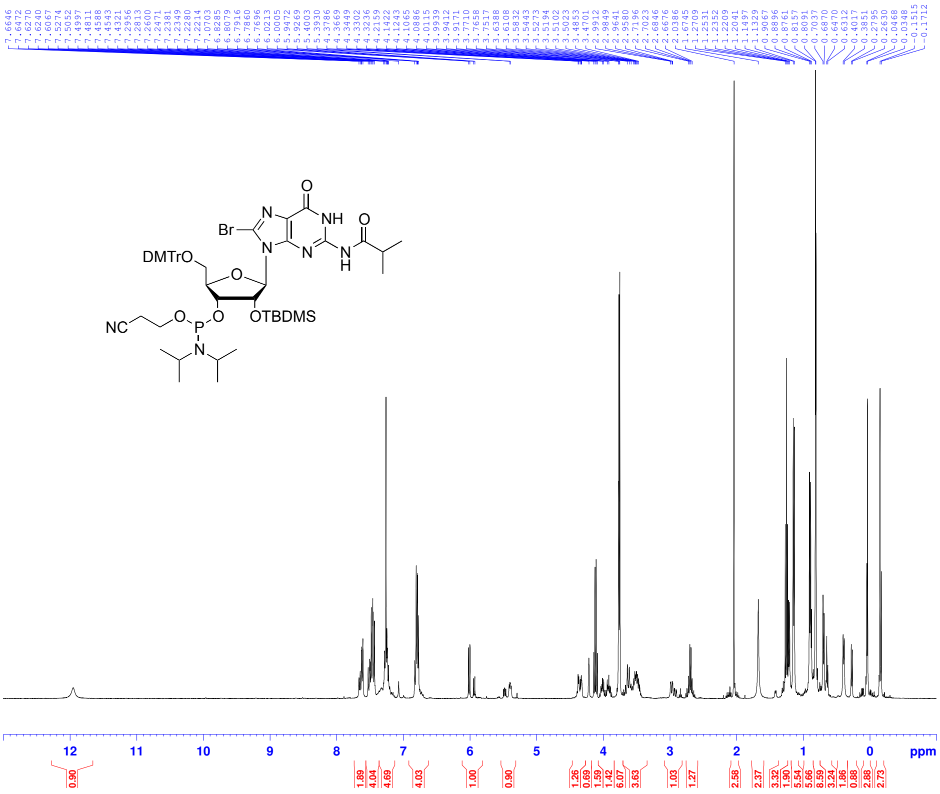

Figure S4. ^{1}H NMR spectrum of compound **5**.

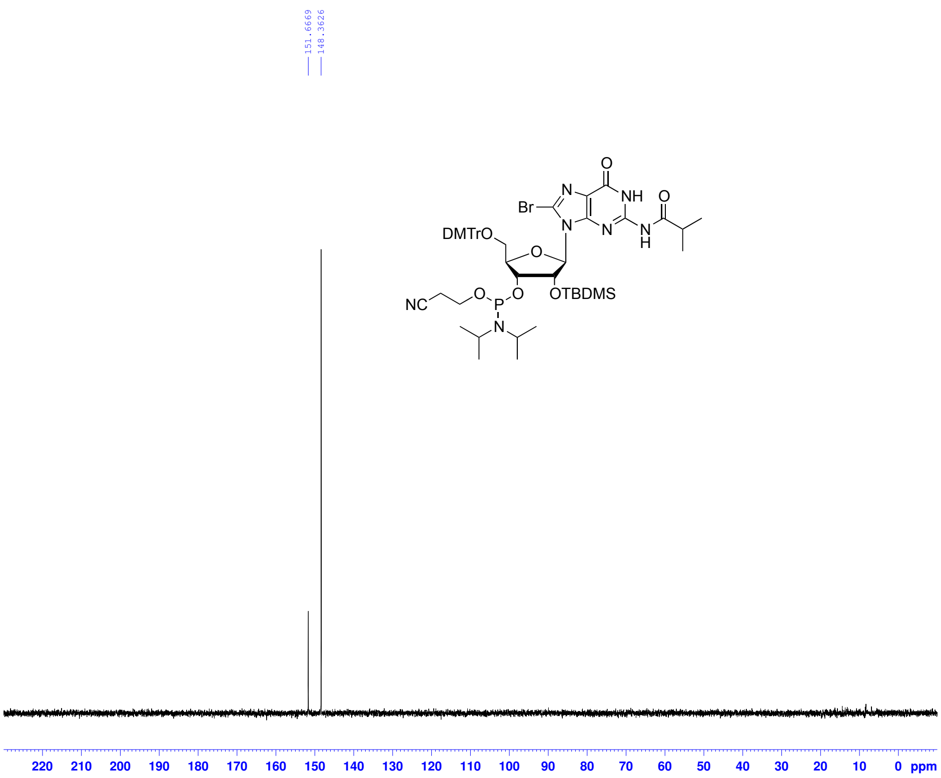

Figure S5. ^{31}P NMR spectrum of compound **5**.

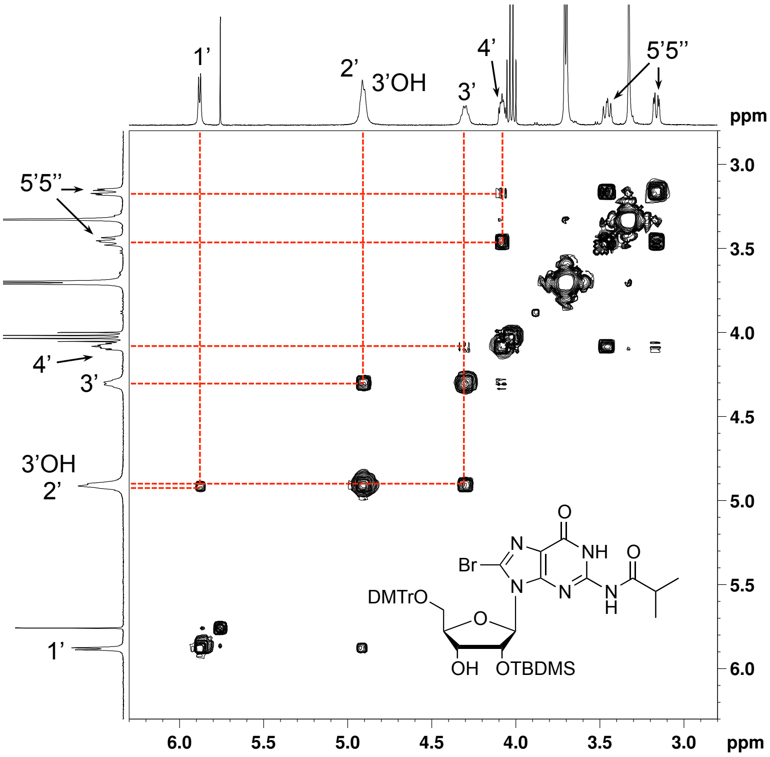

Figure S6. ^{1}H-^{1}H COSY of compound **4**.

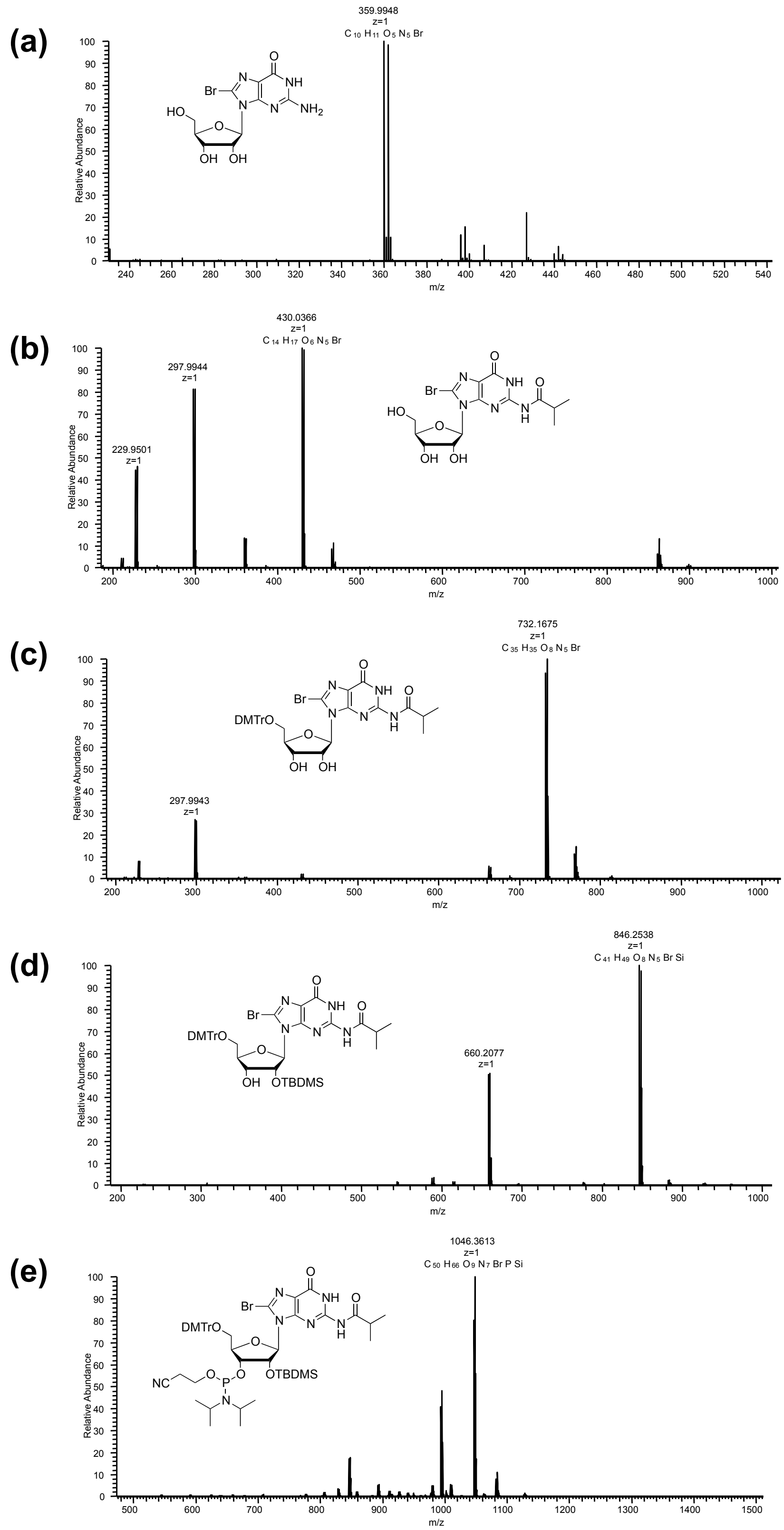

Figure S7. High-resolution mass spectra (HRMS) of all compounds used in this study.

ORN	Sequences	Calcd MS	Found MS
ORN1	5’-UA(8^{Br}rG)GGU-3’	1992.11	1992.15
ORN2	5’-UAG(8^{Br}rG)GU-3’	1992.11	1990.98
ORN3	5’-UAGG(8^{Br}rG)U-3’	1992.11	1990.93

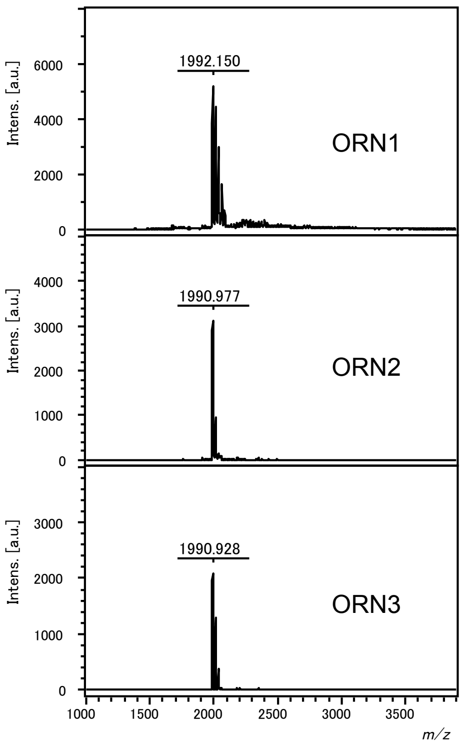

Figure S8. MALDI-TOF MS of ORNs used in this study.

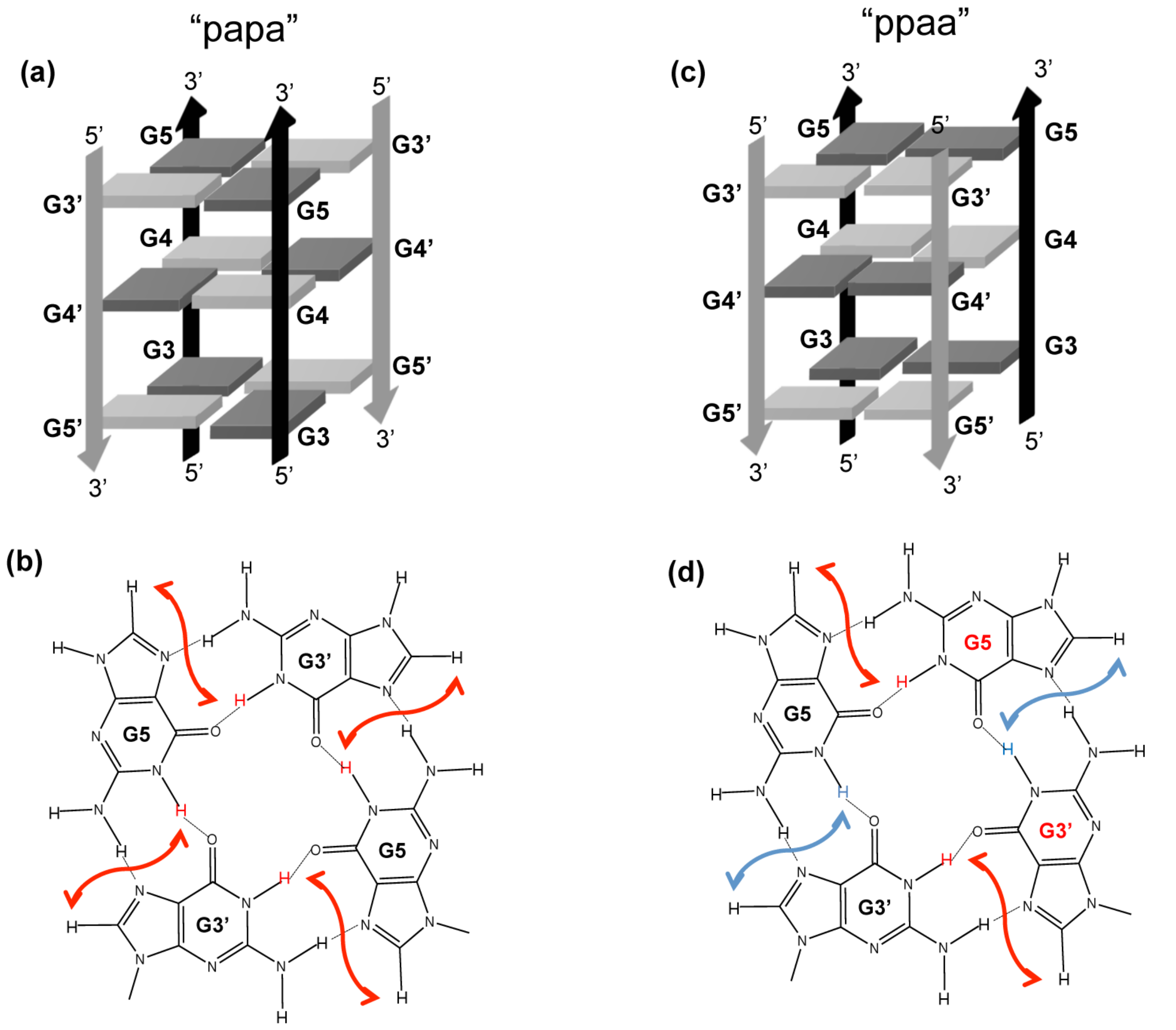

Figure S9. Schematic two possible patterns of G-stem orientation of antiparallel G-quadruplex. Syn and anti residues are in grey and black, respectively. (**a**) “papa” pattern. (**b**) Schematic representation of imino proton of G3’-G5-G3’-G5 tetrad. (**c**) “ppaa” pattern. (**d**) Schematic representation of imino proton of G3’-G3’-G5-G5 tetrad. Two G3’ imino protons at the G3’-G3’-G5-G5 tetrad are not equivalent in “ppaa” structure: the imino proton from G3’ in black connects the G3’ (red), while the imino proton from G3’ in red connects the G5. Two G5 imino protons at the G3’-G3’-G5-G5 tetrad are not equivalent also. This leads to four imino peaks at one tetrad, resulting in twelve imino proton peaks for the structure. This contradicts six well-defined signals observed in the imino proton region of the ^{1}H NMR. In “papa” pattern, two G3’ and G5 imino protons are equivalent in one tetrad, respectively, resulting in six imino proton peaks.

(a)

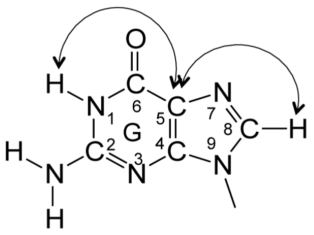

(b)

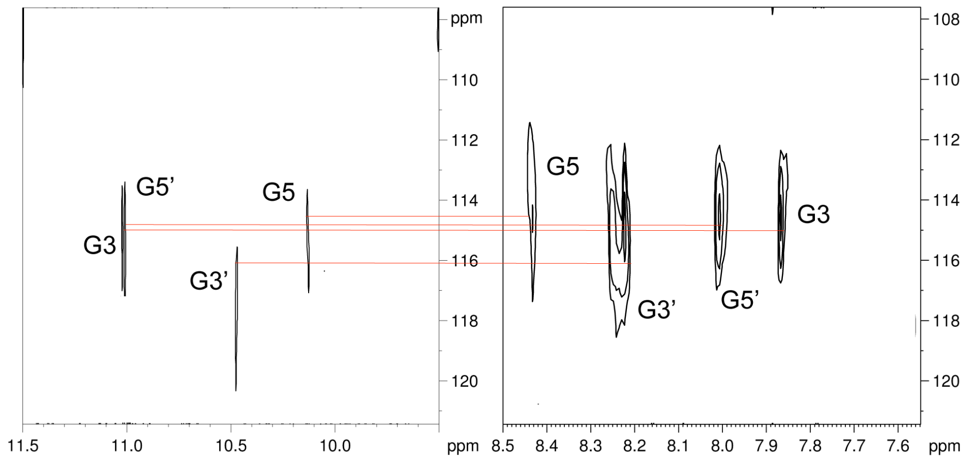

Figure S10. (a) The bases of rG. Arrows indicate the coherence transfer pathways used in the experiments. Long-range J-couplings between imino and H8 protons via ^{13}C5 for rG. (b) JR-HMBC spectra for ORN-2. Through-bond correlations between imino and H8 protons via ^{13}C5 for rG is at natural abundance. The corresponding peaks are labeled with residue numbers. G4 and G4' are absent due to the replacement of H by Br at C8 position. For the JRHMBC spectra 2048 scans per FID, τ = 50 ms, total measurement time of 120 h.

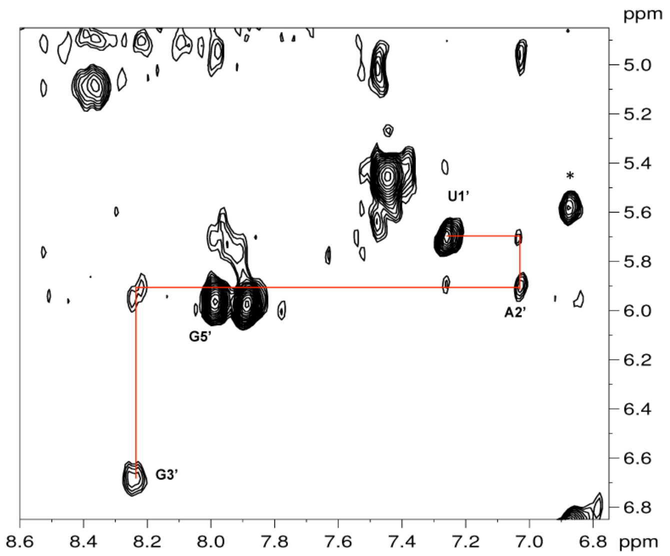

Figure S11. The H8/6-H1' proton region of NOESY spectrum of the ORN-2 G-quadruplex in the presence of 100 mM KCl and 10 mM potassium phosphate buffer. The sequential assignment pathway is shown (red line).

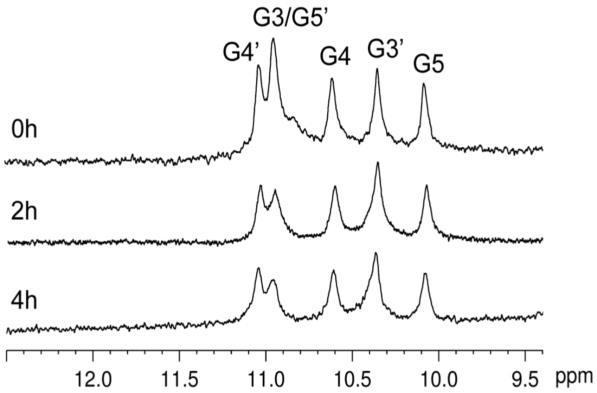

Figure S12. Imino proton region of ^{1}H spectra of ORN-2 G-quadruplex in HDX experiments. G3 and G5' were easily exchanged compared to other G residues. D_2O exchange time is shown. Imino peaks are labeled by numbers

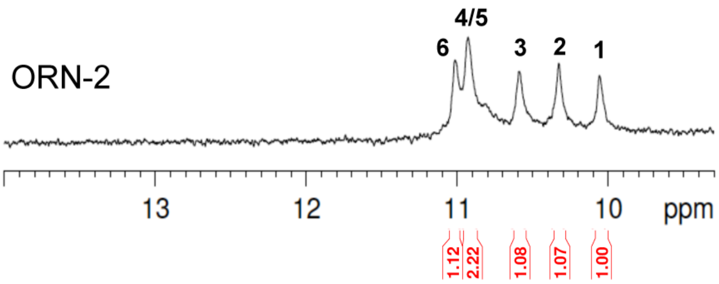

Figure S13. The whole imino proton region of ^{1}H spectrum of ORN-2 in the presence of 100 mM KCl and 10 mM potassium phosphate buffer. Red numbers indicated the relative number of imino protons by the integration of ^{1}H NMR spectrum.

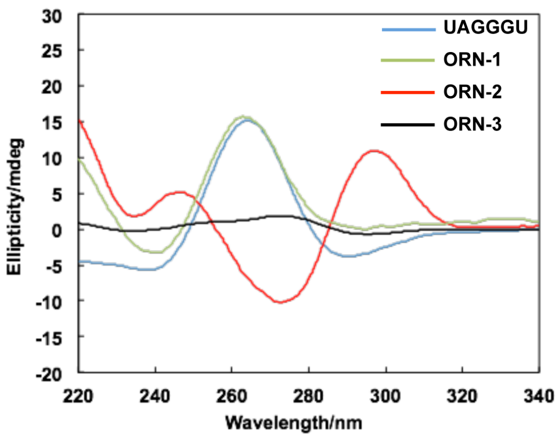

Figure S14. CD spectra of ORN-1, ORN-2, ORN-3 and UAGGGU in the presence of 100 mM KCl at 25 °C.

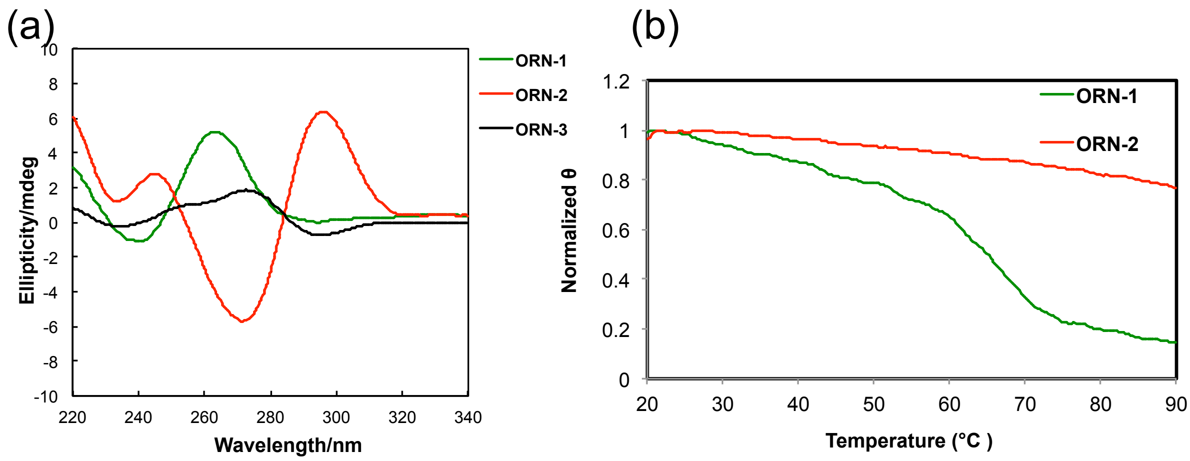

Figure S15. (a) CD spectra of ORN-1, ORN-2 and ORN-3 in the presence of 10 mM KCl at 25 °C. (b) CD melting curves for ORN-1 and ORN-2 monitored at 265 and 295 nm in the presence of 10 mM KCl, respectively.

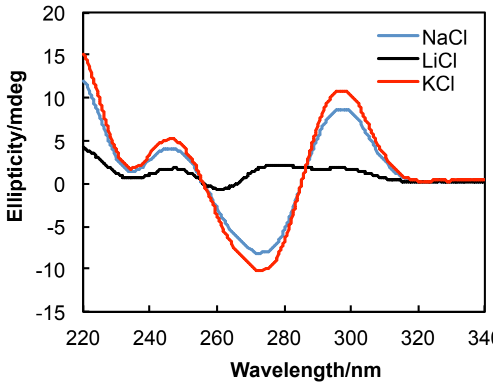

Figure S16. CD spectra of ORN-2 in the presence of 100 mM KCl, 100 mM NaCl and 100 mM LiCl at 25 °C.

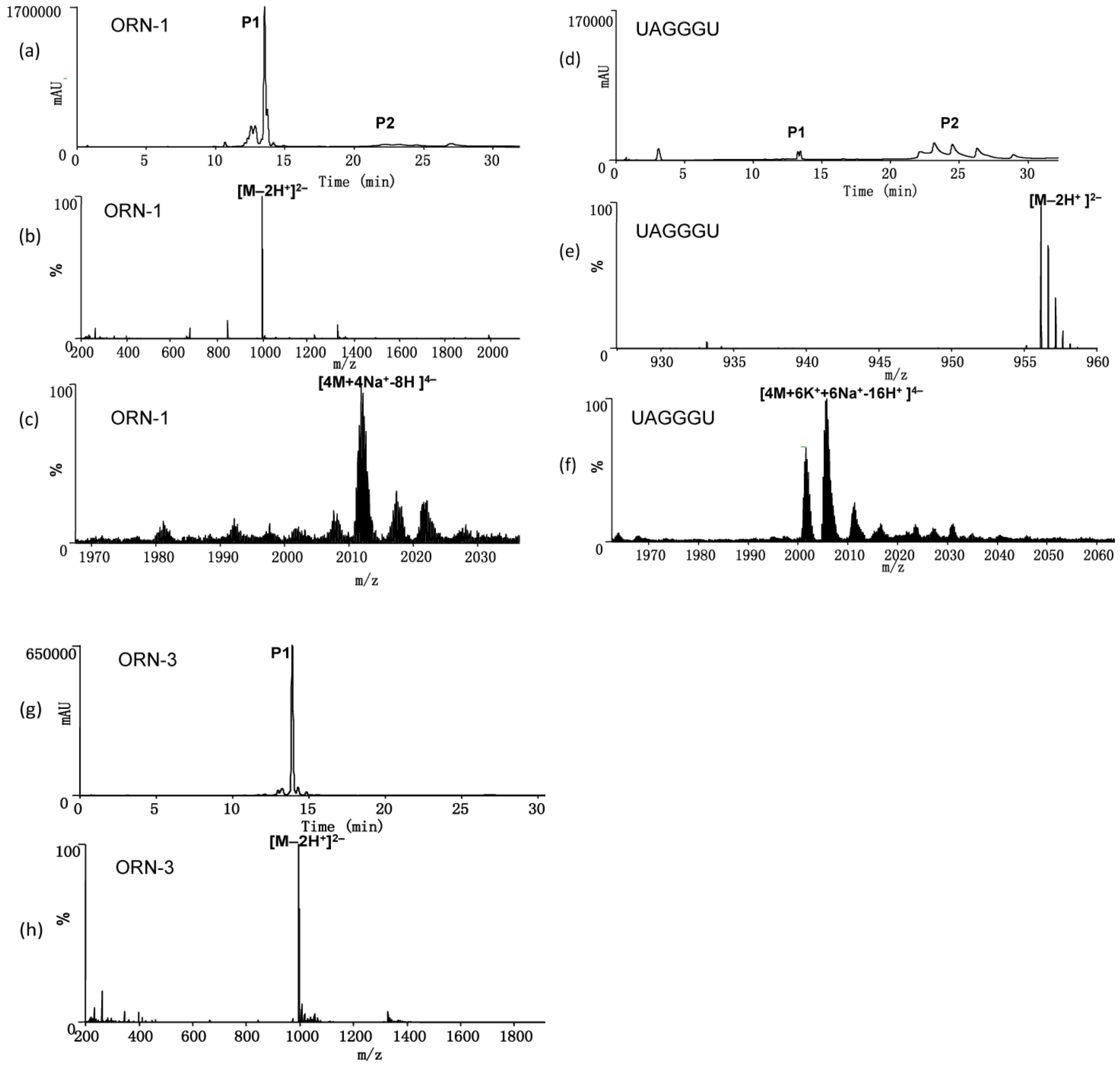

Figure S17. (a) LC analysis result of ORN-1. UV absorbance is monitored at 254 nm. (b) ESI-MS spectrum of P1 peak of ORN-1 in LC. (c) ESI-MS spectrum of P2 peak of ORN-1 in LC. Molecular ions (4^-) are directly observed for G-quadruplex by electrospray ionization MS. Sample concentration is 0.05 mM. M: molecular weight. (d) LC analysis result of r(UAGGGU). (e) ESI-MS spectrum of P1 peak of UAGGGU in LC analysis result. (f) ESI-MS spectrum of P2 peak of UAGGGU in LC. Molecular ions (4^-) are also directly observed for G-quadruplex formed by UAGGGU. M: molecular weight (1914.2). (g) LC analysis result of ORN-3. (h) ESI-MS spectrum of P1 peak of ORN-3 in LC. Only the single strand molecular weight was observed.

MALDI-TOF MS. The purified oligonucleotides were identified by a matrix-assisted laser desorption/ionization-time-of-flight mass spectrometer (MALDI-TOF MS) in a negative mode. The matrix for MALDI-TOF MS was 1:1 mixture of 3-hydroxypicolinic acid (3HPA) in 1:1 acetonitrile/H_2O saturated solution and 0.5 M ammonium citrate aq. solution. 1 μL of DNA sample was mixed with 1 μL of matrix solution. A spot of 1 μL of the sample-matrix mixture was placed on a MALDI target plate (MTP 384 ground steel, Bruker) and allowed to air dry at room temperature. The spectrum was measured using a MALDI-TOF MS on Bruker autoflex II mass spectrometer with dT_5 ($[M-H]^-$: 1458.012) and dT_8 ($[M-H]^-$: 2370.603) as an external calibration standard.

ESI-MS. High-resolution electrospray ionization (ESI) mass spectra were recorded using Exactive Orbitrap mass spectrometer (Thermo Scientific) in negative ion mode. Data was acquired using Xcalibur software (Thermo Scientific). The source conditions maintained were; sheath gas flow rate, 15 psi; aux gas flow rate, 3 psi; capillary temperature, 275 °C; capillary voltage, -32.5 V; tube lens voltage, -165 V; skimmer voltage, -46 V; heater temperature, 45 °C. Scanning parameters were; higher energy collisional induced dissociation (HCD) gas, on; resolution, ultra high; microscans, 1; lock masses, off, AGC target, balanced and maximum injection time, 250 ms. All samples were dissolved in methanol (LC-MS grade, Wako), and the sample solutions were infused into the ESI source at a flow rate of 20 μL/min by using instrument's syringe pump.

Reference

[1] Proctor, D. J.; Kierzek, E.; Kierzek, R. P.; Bevilacqua, C. *J. Am. Chem. Soc.* **2003**, *125*, 2390.
